# P-867. Carbapenem-resistant gram-negative bacterial infections in South India: A four-year retrospective study

**DOI:** 10.1093/ofid/ofae631.1058

**Published:** 2025-01-29

**Authors:** Suresh Kumar Dorairajan, Ratheesh Rajakumar

**Affiliations:** Apollo hospital, Chennai, Tamil Nadu, India; SRM Medical College Hospital & Research Centre , chennai, Tamil Nadu, India

## Abstract

**Background:**

Carbapenem-resistant Gram-negative bacteria (CRGNB) posed a global healthcare threat due to antibiotic resistance. In India, carbapenem resistance surged, and Ceftazidime–avibactam (CAZ-AVI) and Aztreonam (AZT) saw increased use without efficacy data. Previous international studies comparing polymyxins with CAZ-AVI plus AZT highlighted the need for region-specific evaluation. Here, we report the epidemiological, clinical, and treatment outcomes of patients admitted with CRGNB infections in a South Indian tertiary care referral hospital.

Common CRO'S reported during the study period

**Figure:**
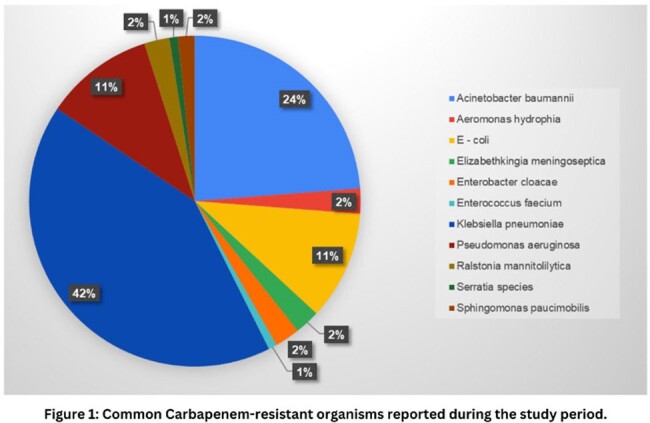

**Methods:**

This retrospective study was conducted at a 250-bed tertiary care referral hospital in South India. Over the last four years (2019 to 2023), we identified all blood culture results indicating the growth of carbapenem-resistant Gram-negative bacteria (CRGNB). Subsequently, we systematically reviewed the medical records of these patients, focusing on demographical, clinical, microbiological, and outcome data. Blood cultures utilized the BacT/ALERT (Biomerieux) culture system, and species identification was accomplished with the VITEK GNI card (Biomerieux). Antibiotic susceptibility testing followed the revised Clinical and Laboratory Standards Institute performance standards. Data were entered into an Excel spreadsheet, and descriptive statistics were employed for data interpretation.

**Results:**

The study comprised 122 patients with positive blood cultures for carbapenem-resistant Gram-negative bacteria (CRGNB), with 70.50% males and 29.50% females, averaging 58.3 years. Common CRGNBs were identified, as shown in Figure 1. Minimum Inhibitory Concentrations (MICs) for colistin were available on 34.4% occasions. CRGNBs exhibited a 41% mortality rate. Combinational therapy (n=79) for CR showed lower mortality than monotherapy (n=43), especially Colistin monotherapy (72% mortality) and Polymyxin B (20%) compared to ceftazidime avibactam + aztreonam (14%) mortality.

**Conclusion:**

A 41% mortality rate was noted in patients with CRGNB infections in South India, with significantly higher mortality (72%) associated with colistin monotherapy. Future strategies should focus on the role of combination therapies to enhance patient outcomes in the context of CRGNB infections.

**Disclosures:**

**All Authors**: No reported disclosures

